# Investigating the optimistic *in-vitro* and *in-vivo* therapeutic effects of wild grape: *Vitis jacqumantii* R. Parker

**DOI:** 10.1016/j.heliyon.2024.e40804

**Published:** 2024-11-30

**Authors:** Tour Jan, Syed Wadood Ali Shah, Nasrullah Khan, Mohammad Sohail Ahmad, Ibrahim A. Saleh, Mohammad K. Okla, Mostafa A. Abdel-Maksoud, Abdullah A. AL-ghamdi, Yasmeen A. Alwasel, Hamada AbdElgawad

**Affiliations:** a Department of Botany, University of Malakand, Dir (L), Khyber Pakhtunkhwa, Pakistan; b Department of Pharmacy, University of Malakand, Dir (L), Khyber Pakhtunkhwa, Pakistan; c Faculty of Science, Zarqa University, Zarqa, Jordan; d Department of Botany and Microbiology, College of Science, King Saud University, Riyadh, Saudi Arabia; e Integrated Molecular Plant Physiology Research, Department of Biology, University of Antwerp, 16 Antwerp, Belgium

**Keywords:** *V. jacqumantii*, Antioxidant, Enzyme inhibition, Analgesic, Antidepressant

## Abstract

*Vitis jacquemontii* R. Parker is a wild grape traditionally used by indigenous people as a substitute for cultivated grapes. However, its therapeutic effects have not been extensively studied. In this study, we investigated the antioxidant, anticholinesterase, analgesic, and antidepressant properties of *V*. *jacquemontii*. The antioxidant potential of this wild fruit plant was evaluated using two widely recognized assays: 2,2-diphenyl-1-picrylhydrazyl (DPPH) and 2,2-asino-bis (3-ethylbenzothiazoline-6-sulfonic acid) (ABTS). *In-vitro* anticholinesterase effects were determined by assessing butyrylcholinesterase (BChE) and acetylcholinesterase (AChE) inhibition. The analgesic activity was assessed through writhing and tail immersion test models, while the antidepressant effect was evaluated using forced swimming and tail suspension test models. Results revealed the exceptional potential of *V*. *jacquemontii* as a valuable natural resource. The fruit extract (VJF-Crd) demonstrated remarkable free radical scavenging abilities, with an impressive IC_50_ value of 34.96 μg/mL for DPPH and 56.48 μg/mL for ABTS. The leaf extract (VJL-Crd) also exhibited considerable antioxidant properties, with IC_50_ values of 73.68 μg/mL for DPPH and 86.72 μg/mL for ABTS. Furthermore, VJF-Crd and VJL-Crd extracts displayed potent inhibitory activity against cholinesterase enzymes, with VJF-Crd demonstrating strong inhibition and VJL-Crd showing moderate inhibition. In terms of analgesia, these extracts exhibited dose-dependent responses in various pain models, with significant protection against acetic acid-induced writhing and tail immersion, showcasing their potential as natural pain relievers. Moreover, both VJF-Crd and VJL-Crd extracts displayed a notable decrease in immobility in the forced swimming and tail suspension test models, indicating their potential as natural antidepressants. These findings underscore the untapped potential of *V*. *jacquemontii* as a source of valuable chemical constituents. The isolation and identification of phyto-constituents from this plant hold promise for new bioactive compounds, particularly in pain management. This study sheds light on the multifaceted medicinal attributes of *V*. *jacquemontii* and opens new avenues for developing natural remedies for different ailments, especially pain management.

## 1 Introduction

The Vitaceae, also known as the grape family, is found in various parts of the world, encompassing approximately 16 genera and 950 species [1]. In Pakistan, it is represented by 6 genera and 12 species [2]. Several species of Vitaceae are utilized as diuretics and in tumors, splenopathy, neuralgia, and leucorrhea astringent treatment [3]. The biological activities and chemical configuration of the fruit and seed of different grapes have been extensively investigated [4]. The fruit of *Vitis vinifera* is a source of wine, grapes, and raisins [5]. The therapeutic potential of the muscadine grape (*Vitis rotundifolia*) can be credited to its copious and varied phytochemicals [6]. The unique phytochemical composition of muscadine grape extract helps prevent cancer growth [7]. The *Cayratia trifolia* fruit and bark extracts possess antiviral, antibacterial, antiprotozoal, and anticancer activity [3]. Many wild plants have been explored for their antioxidant potential. Natural antioxidants either in the form of crude extract or their biochemical ingredients are very active in inhibiting the damaging effects produced by oxidative trauma [8]. *Salvia moorcroftiana* crude extracts effectively combat DPPH free radicals [9]. Free radicals in the body are biochemical responses, involved in inflammation, atherosclerosis, cancer, aging, diabetes, and neurological disorders [10]. To hostage free radicals as proteins, the human body has a blockade system, i.e. catalase, glutathione peroxidase, and superoxide dismutase. Natural phytonutrients are antioxidants, used as defense medicine to combat free radicals inside the living body [11]. Epidemiological research has shown that uninterrupted intake of natural antioxidants is linked with a lesser threat of cancer and heart disease [12,13].

The intake of cholinesterase (i.e. BChE and AChE) preventer medicines, is the top desired therapeutic method to control neurological illnesses [4]. In Alzheimer's disease, the key enzymes are BChE and AChE. The inhibition of these enzymes increases communication and decreases the symptoms of Alzheimer's illness [14]. Extracts from fifteen native New Zealand medicinal plants have the potential to be used as substitute treatments for the typical therapeutics of Alzheimer's disease [15]. The plant constituents are important in decreasing the development of Alzheimer's disease by inhibiting BChE and AChE [16]. Extracts from eight Bangladeshi medicinal plants have been utilized to treat Alzheimer's and other neurodegenerative diseases [17]. The uses of synthetic drugs in medication for Alzheimer's disease have adverse side effects on human health [18,19]. Recent studies, on antialzheimer and anti-diabetic, natural compounds in herbal sources and food have been amplified [20,21]. Natural compounds have various properties, including the anti-enzymatic properties of enzymes such as α-glucosidase, α-amylase, tyrosinase, BChE, and AChE [22]. They can be achieved from wild plants [23] and honey products [24]. The mechanism of interaction and action of these compounds is very complicated and depends on the nature of the target and action. Moreover, studies should not only aim to investigate antioxidant and enzyme inhibition but should also be varied to achieve all the therapeutic effects. One of the investigated activities in plant species is analgesic activity.

Pain is an inactivating sensation that indicates something is wrong in the body. Painkillers are an important therapeutic priority [25]. Nonsteroidal and opiate anti-inflammatory drugs are used to treat or reduce pain and are based on natural resources. Synthetic compounds have been developed that work similarly and are associated with negative effects [26,27]. Synthetic compounds have negative effects, and the common negative effects of synthetic compounds are bleeding, ulcers, and renal disorders [28]. Plants are the major source of medicinally active phytochemical constituents with reduced negative side effects [29]. The extract from *E. cymosa* showed significant analgesic and anti-inflammatory properties, confirming its traditional use as a remedy for various painful and inflammatory conditions [30]. Depression illness is prevalent worldwide [31,32], the world health organization has reported that depression is a severe mood disorder that upsets millions of the world population [33]. These factors indicate the need to search and discover new biochemical compounds from natural resources, particularly wild plants, as alternatives for analgesics and depression treatment with minimal to no adverse effects. This study aims to evaluate the biological activities mainly antioxidant, enzyme inhibition (i.e. cholinesterase), analgesic and antidepressant potential of fruit and leaf extracts of *V. jacquemontii* R. Parker. There is a dire need to make a breakthrough in research, as there is no study in the literature.

## 2 Materials and methods

### 2.1 Experimental materials

Leaves (4 kg) and fruits (0.5 kg) of *V. jacquemontii* were collected in July 2019 and dried in shade in a ventilated place at room temperature. Upon drying, the leaves and fruits were ground to powder. The dried powder was dipped separately in 85 % methanol for 10 days. The resulting suspensions were filtered and the filtrates were subjected to a rotary evaporator for solvent separation. The formula calculated the percentage yield of extracts:

$$Yield\;\%=\frac{The\;dry\;weight\;of\;extract}{The\;dry\;weight\;of\;plant\;powder}\times 100$$

The methanolic extracts were then tested for antioxidant, and enzyme inhibition i.e. cholinesterase, analgesic, and antidepressant activities.

### 2.2 Antioxidant inhibitory activity

Antioxidant inhibitory activity of the samples (VJL-Crd and VJF-Crd extracts) was performed using DPPH and ABTS assay following the method of Grzegorczyk and Kiss [34].

To evaluate 2,2-diphenyl-1-picrylhydrazyl (DPPH) free radical scavenging activity, different concentrations of VJL-Crd and VJF-Crd extracts were prepared in methanol (1000, 500, 250, 125, and 62.5 μg/ml). Aliquots of 2 ml of different concentrations of the plant extracts (1000, 500, 250, 125, and 62.5 μg/ml) were mixed with 2 ml of DPPH (100 μM) and incubated for 30 min at 25 °C. A blank solution containing an equal amount of methanol and DPPH radicals was formulated. A spectrophotometer was used to measure the absorbance at 517 nm wavelength.

The 2,2-azino-bis(3-ethylbenzothiazoline)-6-sulfonic acid (ABTS) assay was performed to evaluate the potential effect of VJL-Crd and VJF-Crd extracts against ABTS free radical scavenging effect using the methodology of [35]. Briefly, the radical cation was formulated by homogenizing ABTS (7 mM) and potassium persulphate (2.45 mM) and kept the homogenizer for 16 h. Various concentrations of VJL-Crd and VJF-Crd extracts were prepared (1000, 500, 250, 125, and 62.5 μg/ml). ABTS solution of 3.9 ml was supplemented with 0.1 ml of each extract. After adding the standard and each extract, absorbance was measured each minute for 30 min, at the wavelength of 734 nm. The DPPH and ABTS results were expressed as IC_50_ (μg/mL).

### 2.3 *In vitro* enzyme inhibition

Anticholinesterase potentials of VJL-Crd and VJF-Crd extracts were determined, using butrycholinestrerase (BChE) and acetylcholinesterase (AChE) enzymes. In test tubes, different mixtures of samples (VJL-Crd and VJF-Crd) (50 μL) and enzymes (BChE and AChE) (0.5 mL) were taken and kept warm at 25 °C. In these test tubes buffer of 2.4 ml and DTNB 100 μL were added, and kept warm again at 25 °C for 5 min. The reaction was initiated, with the supplementation of 40 μL ATChI, and kept warmed for 20 min at 25 °C. The AChE and BChE inhibitory potentials of various dilutions of test samples were evaluated using the reagent BuChE enzyme, DTNB, and BTChI, and mixed as per the details above. At wavelength 412 nm, the absorbance was measured with a spectrophotometer. The data was repeated three times and IC_50_ was calculated [36].

### 2.4 Animals and ethical approval

*In vivo* biological activities of the extracts were performed using healthy Balb/C mice of both sexes (male and female), aged 4 weeks and weighing 20–25 g. Animals were acquired from the National Institute of Health (NIH), Islamabad, Pakistan, and housed in an animal house in standard laboratory environments, providing standard food and water (*ad libitum*). The biological activities were executed according to standard methods, at the Department of Pharmacy, University of Malakand, Pakistan. After conducting tests, the mice were lost by euthanasia with isoflurane. All procedures used in this research were permitted by the Departmental Ethical Committee (Pharm/EC-Vg/24-02/21) by the University of Malakand Animal Bye-Laws 2008, Scientific Procedures Issue-I.

### 2.5 Acute toxicity

The toxicity of VJL-Crd and VJF-Crd extracts was evaluated on mice at various doses following the protocol of [37]. Doses were administered orally and the mice were continuously examined for signs of indications of convulsions, diarrhea, lethargy, salivation, sleeping, and mortality. The investigational mice were distributed into 6 groups of 8 mice in each group:

Group 1: Control.

Group 2: Positive control.

Group 3: Test group- VJL-Crd extract100 mg/kg intraperitoneally).

Group 4: Test group- VJL-Crd extract 200 mg/kg intraperitoneally).

Group 5: Test group- VJF-Crd extract100 mg/kg intraperitoneally).

Group 6: Test group- VJF-Crd extract 200 mg/kg intraperitoneally).

The control (group-1) received tween-80 (2 %) as the vehicle. Positive control (Group 2) received 10 mg/kg diclofenac sodium for acetic acid-induced writhing and tramadol (20 mg/kg) for tail immersion. The selection of doses of extracts was based on the preliminary pharmacological activity. The test groups (3–6) received 100 and 200 mg/kg of the extracts.

### 2.6 Analgesic activity

According to the following two models, the analgesic experiments of the VJL-Crd and VJF-Crd extracts were conducted.

#### 2.6.1 Acetic acid-induced writhing test model

An acetic acid-induced writhing test was conducted according to the procedure of [38]. Acetic acid was administered to the mice intraperitoneally to induce pain sensitivity. The samples and standard were given intraperitoneally to the experimental mice. 0.1 ml of 0.7 % acetic acid was injected intra-peritoneally, following the administration of diclofenac sodium 15 min before acetic acid injection. After injection of acetic acid, then the mice were retained on an observation table and the number of writhing movements was counted for 15 min beginning 5 min. The number of writhes in the groups treated with extracts was matched with control whereas 10 mg/kg diclofenac sodium was used as a positive control.

#### 2.6.2 Tail immersion test model

The tail immersion experiment was executed according to the procedure of [39]. Animals were distributed in four groups of 8 animals. The test was carried out by calculating the time it took for the mice to flick their tails away from hot water (55 ± 2 °C). After that, 10 ml/kg of the extracts were supplied to the mice and data were noted. Before the tail immersion test tramadol 20 mg/kg was administered for 30 min.

### 2.7 Antidepressant activity

Antidepressant activity of the VJL-Crd and VJF-Crd extracts was performed using forced swimming and tail suspension test models.

#### 2.7.1 Forced swimming test model

The forced swimming test (FST) experiment was performed according to the method of [40]. Mice were forced to swim individually in a glass jar (height 30 cm and diameter 15 cm) comprising 20 cm of fresh water (25 ± 1 °C). Extracts of the VJL-Crd and VJF-Crd were given to mice at doses of 100 and 200 mg/kg 30 min before the testing session. Mice were forced to swim and the time length of immobility was calculated in the final 4 min of the 6 min testing session. A mouse was decided to be motionless when it stopped struggling and kept moving immobile in the water, considering only those movements required to keep its head above water. Reduction in the period of immobility during the FST is symptomatic of an antidepressant-like effect.

#### 2.7.2 Tail suspension test model

The tail suspension test (TST) experiment was performed according to the method of [41]. Extracts of the VJL-Crd and VJF-Crd were given to the mice at 100 and 200 mg/kg, 30 min before the testing session. Depression was induced by hanging the mice through an adhesive tape placed nearly 1 cm from the tip of the tail, above 50 cm from the floor. After 3–4 min of strong activity, such as attempts to catch the glue tape, body rotation, or jerking movements, a mouse was considered motionless when it displayed no body activity and remained completely immobile. The period of immobility was measured for 6 min of the total 10 periods. The antidepressant effect manifests a decrease in immobility time between the control, standard, and test extracts [42].

### 2.8 Statistical analysis

We computed descriptive statistics (mean and standard error; Mean ± SEM), to summarize the central tendency and variability of the experimental results. We performed a one-way analysis of variance (ANOVA) to determine statistically significant differences. This statistical technique is suitable for the comparison of means across multiple independent treatments, confirming that any significant findings reflect true differences in the experimental data rather than random variation. Subsequently, Dunnett's post hoc test was performed to detect specific group differences. All the analysis was carried out using GraphPad Prism version 5.01 (GraphPad Prism Software, Inc., San Diego, CA, USA). Additionally, correlation coefficient (Pearson's r) was calculated for assessing the relationship between radical scavenging activity (DPPH and ABTS assays) and cholinesterase inhibitory activity (AChE and BChE assays) in VJL and VJF samples across different concentrations. This analysis was performed using OriginPro software (Ver 24."

## 3 Results

### 3.1 DPPH and ABTS scavenging activities

**Fig. 1:**
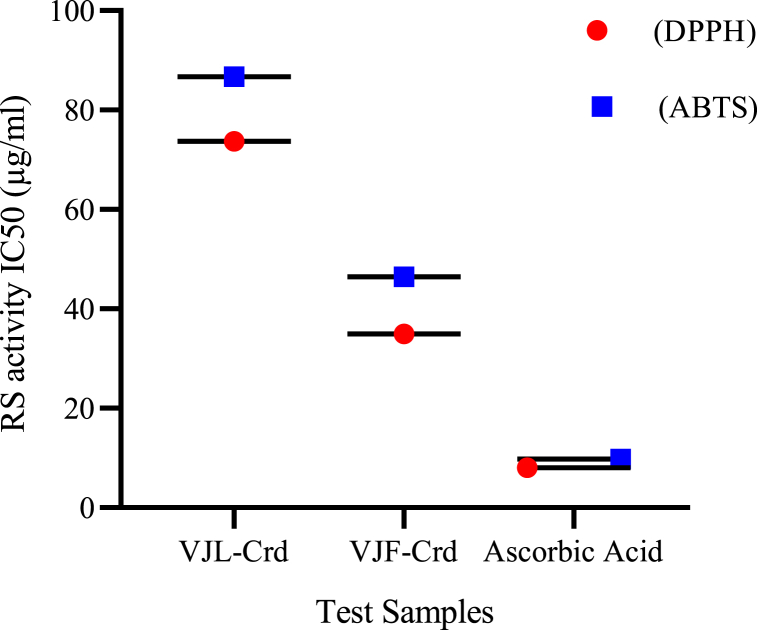
Potency of the methanol extracts of *V. jacquemontii* against DPPH and ABTS assay.

In this study, we employed *in vitro* DPPH and ABTS assays to assess the antioxidant potential of VJL-Crd and VJF-Crd extracts from *V. jacquemontii*. Fig. 1 illustrates that VJF-Crd extract exhibited greater potency than VJL-Crd extract in both DPPH and ABTS scavenging tests. The IC_50_ values for DPPH were 34.96 μg/mL and 73.68 μg/mL, and for ABTS were 86.72 μg/mL and 46.48 μg/mL for VJF-Crd and VJL-Crd, respectively. Notably, standard ascorbic acid surpassed both extracts with IC_50_ values of 8.07 μg/mL for DPPH and 9.79 μg/mL for ABTS (Fig. 1).

### 3.2 *In vitro* enzyme inhibitory effect

**Fig. 2:**
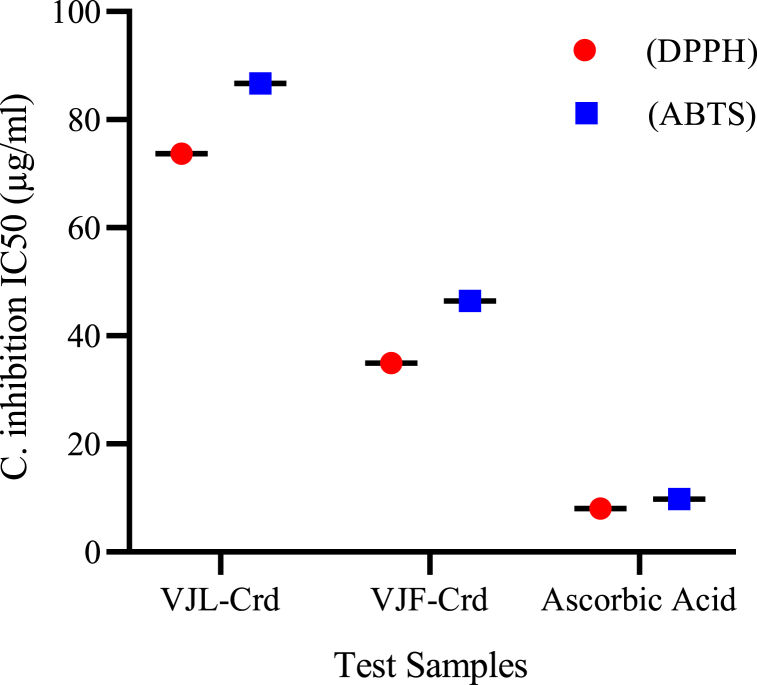
Inhibitory potency of the methanol extracts of *V. jacquemontii* against cholinesterase.

Moving on to the anticholinesterase properties, both VJL-Crd and VJF-Crd extracts exhibited inhibition of AChE and BChE. Fig. 2 compares the cholinesterase inhibitory properties of VJL-Crd, VJF-Crd extracts, and the reference standard (Donepezil). VJF-Crd extract demonstrated significantly higher AChE inhibition compared to VJL-Crd. The IC50 values for AChE were 52.88 μg/mL and 84.63 μg/mL, and for BChE were 57.11 μg/mL and 89.73 μg/mL for VJF-Crd and VJL-Crd, respectively. However, Donepezil exhibited greater potency with IC50 values of 4.71 μg/mL for AChE and 3.65 μg/mL for BChE (Fig. 2).

### 3.3 Correlation analysis of inhibition and concentrations for antioxidants and enzymes

**Fig. 3:**
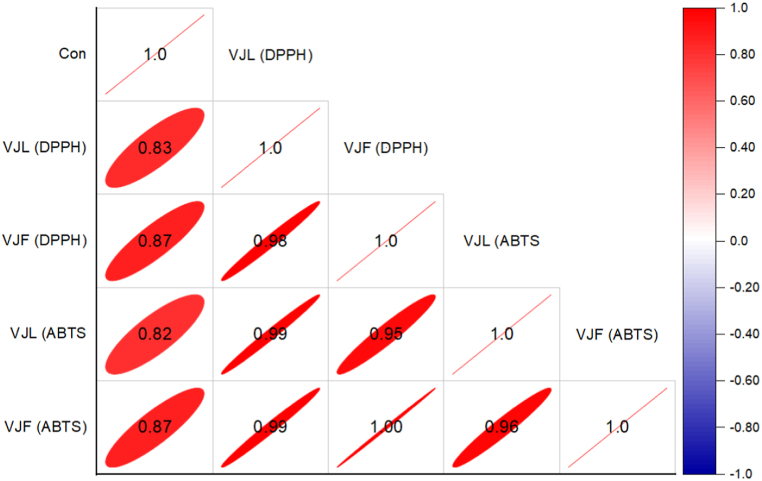
Correlation analysis between radical scavenging activity (DPPH and ABTS assays) in VJL and VJF samples across different concentrations.

**Fig. 4:**
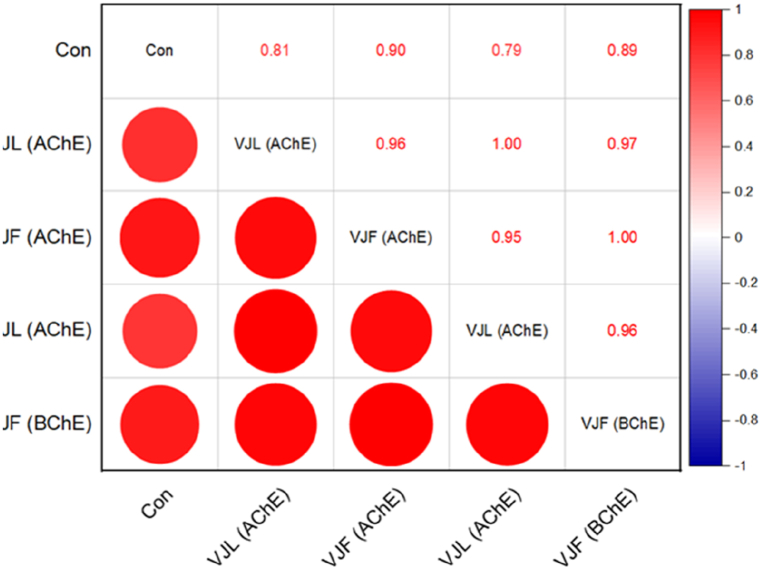
Relationship between cholinesterase inhibitory activity (AChE and BChE assays) in VJL and VJF samples across different concentrations.

The correlation analysis between radical scavenging activity (DPPH and ABTS assays) and varying concentrations in VJL and VJF samples is presented in Fig. 3. The results indicate that DPPH activity increases significantly with higher concentrations in both VJL (*r* = 0.83, *p* < 0.001) and VJF (*r* = 0.87, *p* < 0.001) samples. A similar trend was observed in the ABTS assays for VJL and VJF samples (Fig. 3). Additionally, the cholinesterase inhibitory activity (AChE and BChE assays) in these samples also exhibited strong linear correlations across different concentrations, with these relationships being even more pronounced than those observed for radical scavenging activities (Fig. 4). For instance, the correlation between VJL (AChE) and VJF (AChE) assays accounted for 90 %–96 % of the variance, underscoring the significant impact of increasing concentrations.

### 3.4 Acute toxicity test

The application of VJL-Crd and VJF-Crd extracts showed no mortality, irritation, or behavioral signs in mice at doses up to 200 mg/kg (b.w), indicating their non-toxic nature.

### 3.5 Analgesic effect

The analgesic effect was evaluated through acetic acid-induced writhing and tail immersion test models (Table 1, Table 2), demonstrating inhibition of pain behavior for both extracts. Notably, VJF-Crd extract exhibited higher inhibition percentages than VJL-Crd, though slightly lower than Diclofenac. Both VJL-Crd and VJF-Crd extracts of *V. jacquemontii* exhibited inhibition of pain behavior. In the acetic acid-induced writhing, after oral administration of two different doses of extracts (100 and 200 mg/kg bw), the number of writhing in the control group was 60.21 ± 0.98 (Table 1). Whereas, in the VJF-Crd administered group the number of writhing reduced to 20.43 ± 0.29 and17.13 ± 0.23 with inhibitory percentages of 62.18 and 65.87 %. The number of writhing in the VJL-Crd administered extract groups were 25.11 ± 0.35 and 23.65 ± 0.33 with inhibitory percentages of 50.68 % and 54.26 %, respectively. However, in the Diclofenac (10 mg/kg) administered group, the number of writhing was 9.31 with an inhibitory percentage of 84.55 % (see Table 1).

**Table 1:** Effect of the methanol extracts of *V. jacquemontii* against acetic acid induced writhing on mice.

Sample	Doses (mg/kg)	Number of writhing	Inhibitory%
VJL-Crd	100	25.11 ± 0.35∗∗∗	50.68
	200	23.65 ± 0.33∗∗∗	54.26
VJF-Crd	100	20.43 ± 0.29∗∗∗	62.18
	200	17.13 ± 0.23∗∗∗	65.87
Diclofenac	10	9.31 ± 0.17∗∗∗	84.55
Control	0.0	60.21 ± 0.98	0.0

Mean ± SEM (n = 8). (∗∗∗P < 0.001, n = 8) by *vs* control group using one-way ANOVA following Dunnett's comparison test.

**Table 2:** Effect of the extracts of *V. jacquemontii* against tail immersion test model in mice.

Sample	Doses (mg/kg)	Latency	Inhibition%
VJL-Crd	100	2.19 ± 0.12∗∗	51.14
	200	2.39 ± 0.13∗∗∗	55.23
VJF-Crd	100	3.35 ± 0.21∗∗∗	54.05
	200	3.65 ± 0.24∗∗∗	68.79
Tramadol	20	3.95 ± 0.25∗∗∗	72.91
Control	0.0	1.07 ± 0.15	0.0

Mean ± SEM (n = 8). (∗∗∗P < 0.01, ∗∗∗P < 0.001, n = 8) by *vs* control group using one-way ANOVA following Dunnett's comparison test.

### 3.6 Evaluation of antidepressant effects

In the tail suspension test (TST), VJL-Crd and VJF-Crd extracts induced a dose-dependent reduction in immobility duration, similar to the effect observed with Imipramine. The swimming time in treated mice decreased compared to the control, indicating the potential antidepressant activity of the extracts. The antidepressant properties of VJL-Crd and VJF-Crd extracts from *V. jacquemontii* were assessed by monitoring changes in the duration of immobility during the forced swimming test and tail immersion test.

#### 3.6.1 Forced swimming test

The results in Table 3, indicate that the VJL-Crd and VJF-Crd extracts affected the mice's immobility. Treatment with different doses of VJL-Crd and VJF-Crd extracts, as well as Imipramine, resulted in a significant reduction in the immobility of mice compared to the control group. Both extracts and Imipramine treatments decreased the immobility of the treated mice, with the results for VJF-Crd extract being particularly promising compared to VJL-Crd extract (Table 3).

**Table 3:** Antidepressant activity of the extracts of *V. jacquemontii* in force swimming test.

Sample	Doses (mg/kg)	Immobility (seconds)
Control	0.0	170 ± 0.73
Imipramine	10	70 ± 0.45∗∗∗
VJL-Crd	25	119 ± 0.57∗∗
	50	107 ± 0.42∗∗
VJF-Crd	25	111 ± 0.49∗∗
	50	96 ± 0.33∗∗∗

Mean ± SEM (n = 8). (∗∗∗P < 0.01, ∗∗∗P < 0.001, n = 8) by *vs* control group using one-way ANOVA following Dunnett's comparison test.

#### 3.6.2 Tail suspension test (TST)

During the TST, VJL-Crd and VJF-Crd extracts affected the immobility of mice compared to the standard antidepressant (Imipramine) and control. Administration of VJL-Crd and VJF-Crd extracts led to a dose-dependent reduction in immobility duration in the treated mice (Table 4). Similarly, Imipramine also induced a decrease in immobility time in mice when compared with the control group. In mice treated with VJL-Crd and VJF-Crd extracts, swimming time was reduced compared to the control group (Table 4).

**Table 4:** Antidepressant activity of the extracts of *V. jacquemontii* in tail suspension test.

Sample	Doses (mg/kg)	Immobility (seconds)
Control	0.0	180 ± 0.86
Imipramine	10	85 ± 0.75∗∗∗
VJL-Crd	25	142 ± 0.63∗
	50	127 ± 0.54∗∗
VJF-Crd	25	121 ± 0.42∗∗
	50	103 ± 0.37∗∗∗

Mean ± SEM (n = 8). (∗P < 0.05, ∗∗∗P < 0.01, ∗∗∗P < 0.001, n = 8) by *vs* control group using one-way ANOVA following Dunnett's comparison test.

## 4 Discussion

Antioxidant, anticholinesterase, analgesic, and antidepressant potential of the *V. jacquemontii* has not been evaluated. Therefore, the present data should be assumed as the first report for this plant species. Recently, a natural antioxidant and their health benefits have gained popularity. Antioxidant-based medications are utilized to inhibit and treat a wide range of ailments. The primary source of natural antioxidants is plants, which produce a variety of phyto-constituents with anti-oxidative and therapeutic properties [43,44]. To study the complex nature of neurological disorders such as Alzheimer's disease, antioxidant activities would be of benefit. Therefore, the VJL-Crd and VJF-Crd extracts of *V. jacquemontii* were studied for antioxidant activity to prove their efficacy in neurodegenerative illnesses. In DPPH and ABTS scavenging assays, the VJF-Crd extract exhibited the lowest IC_50_ values compared to the VJL-Crd extract against DPPH and ABTS free radicals in a dose-depending manner. The lower IC_50_ of DPPH and ABTS values mean higher antioxidant activity of the fruit (VJF-Crd extract) of *V. jacquemontii*. These results endorsed the previous result of [45] for determining antioxidant activity in various plants, especially fruit plants. According to literature studies, the antioxidant properties found in plant extracts are mainly attributed to the unique redox properties of various bioactive compounds, rather than just one specific compound [46,47]. The antioxidant effect occurs because the compounds in the plant extract can transfer electrons or hydrogen atoms, which neutralize radicals of DPPH, ultimately forming neutral DPPH molecules [48]. Based on our findings, the study plant may be used as a food enhancement in numerous illness treatments.

We investigated the *in vitro* anticholinesterase properties of the VJL-Crd and VJF-Crd extracts against AChE and BChE enzymes. Both VJL-Crd and VJF-Crd extracts exhibited inhibition against AChE and BChE enzymes. This plant extract's inhibition of AChE and BChE enzymes indicates its potential to preserve brain function and mitigate the risk of Alzheimer's disease. Furthermore, the isolation of natural products from this plant may yield the discovery of new compounds appropriate to control the activities of these enzymes in the human body. Alzheimer's is a neurodegenerative disorder, which is regarded as the decline of mental functions and is directly associated with the damage of cholinergic neurotransmission [49]. AChE and BChE enzymes cause inactivation and hydrolysis of acetylcholine, which functions as a neurotransmitter. The manufactured preventers of BChE and AChE have little therapeutic effects on memory [50].

VJL-Crd and VJF-Crd extracts of *V. jaquemontii* were investigated for analgesic activity using two different models: the writhing test model and the tail immersion test model on mice. The acetic acid-induced writhing test model is the ideal assay for evaluating the antinociceptive potential of drugs or medicinal plants [51]. The acetic acid-induced writhing looks like visceral pain. The writhing induced by the test is characterized by the lengthening of the forelimbs, the enlargement of the body, and the shrinkage of the abdominal muscles [52,53]. In the experimental mice, this enlargement is assumed to be induced by the stimulation of peritoneal receptors and the prostaglandin pathway. The peritoneal muscles may be liable for abdominal writhing [54]. Both extracts of *V. jaquemontii* induced a decrease in writhing and acetic acid extension in mice. The VJF-Crd extract exhibited good inhibition which was neighboring to the diclofenac and higher than the VJL-Crd extract on mice. This analgesic effect of the extracts is indorsed to its phytoconstituents. The present evaluation further demonstrated that VJL-Crd and VJF-Crd extracts of the *V. jaquemontii* were also effective against the tail immersion test model.

Depression is a psychiatric ailment that affects people's quality of life directly and is described by symptoms i.e. loss of self-confidence, sense of guilt, and hopelessness. Medicines such as reversible inhibitors of monoamine oxidase A, selective serotonin reuptake inhibitors, and tricyclic antidepressants are medicine, active for drug therapy [55]. These antidepressant drugs have a diversity of negative effects [56,57]. In the present research, the antidepressant effects of *V. jacquemontii* were assessed using the forced swimming test model and the tail suspension test model. These are commonly recognized behavioral models for evaluating antidepressant effects [58]. Both extracts (VJL-Crd and VJF-Crd) of this plant exhibited that it has an antidepressant effect. The VJF-Crd extract induced a clear decrease in immobility time at higher doses used in the mice FST and TST, which was neighboring to the standard antidepressant drug imipramine. It has been discussed that the forced swimming test (FST) is a less sensitive model for identifying selective serotonin reuptake inhibitors, antidepressants are typically considered to be active in the tail suspension test (TST) [59]. Moreover, TST is measured to be less stressful than FST. Therefore, it is suggested that FST has better pharmacological sensitivity compared to TST [60,61].

**Conclusion**: The present study for the first time indicated that VJL-Crd and VJF-Crd extracts of *V. jacquemontii* exhibited antioxidant and anticholinesterase activities. It also revealed an analgesic and antidepressant activity in various models which may be used as a healthcare fruit supplement and a candidate for pharmaceutical plant-based products. Thus, further intensive study is required to reveal the bioactive compounds within *V. jacquemontii* for the observed therapeutic activities.

## CRediT authorship contribution statement

**Tour Jan:** Writing – original draft, Supervision, Methodology, Formal analysis, Conceptualization. **Syed Wadood Ali Shah:** Methodology, Formal analysis, Data curation. **Nasrullah Khan:** Writing – review & editing, Writing – original draft, Software, Methodology, Formal analysis. **Mohammad Sohail Ahmad:** Writing – original draft, Methodology, Formal analysis, Data curation. **Ibrahim A. Saleh:** Writing – review & editing, Methodology, Investigation, Formal analysis. **Mohammad K. Okla:** Writing – review & editing, Visualization, Validation, Project administration. **Mostafa A. Abdel-Maksoud:** Writing – review & editing, Project administration, Methodology, Formal analysis. **Abdullah A. AL-ghamdi:** Writing – review & editing, Visualization, Methodology. **Yasmeen A. Alwasel:** Writing – review & editing, Software, Investigation, Formal analysis. **Hamada AbdElgawad:** Writing – review & editing, Validation, Resources, Formal analysis.

## Data availability

Data will be made available on request.

## Declaration of competing interest

The authors declare the following financial interests/personal relationships which may be considered as potential competing interests: Nasrullah Khan reports financial support, article publishing charges, and writing assistance were provided by 10.13039/501100002383King Saud University, Riyadh, Saudi Arabia. Nasrullah Khan reports a relationship with University of Karachi that includes: board membership. If there are other authors, they declare that they have no known competing financial interests or personal relationships that could have appeared to influence the work reported in this paper.
